# Nox2 Knockout Delays Infarct Progression and Increases Vascular Recovery through Angiogenesis in Mice following Ischaemic Stroke with Reperfusion

**DOI:** 10.1371/journal.pone.0110602

**Published:** 2014-11-06

**Authors:** Sarah K. McCann, Gregory J. Dusting, Carli L. Roulston

**Affiliations:** 1 Stroke Injury and Repair Team, O'Brien Institute, St Vincent's Hospital, Melbourne, Victoria, Australia; 2 Department of Surgery, Faculty of Medicine, Dentistry and Health Sciences, University of Melbourne, Melbourne, Victoria, Australia; 3 Cytoprotection Pharmacology Program, Centre for Eye Research, the Royal Eye and Ear Hospital, Melbourne, Victoria, Australia; 4 Department of Ophthalmology, Faculty of Medicine, Dentistry and Health Sciences, University of Melbourne, Melbourne, Victoria, Australia; 5 Department of Medicine, Faculty of Medicine, Dentistry and Health Sciences, St Vincent's Campus, University of Melbourne, Melbourne, Victoria, Australia; University of Iowa, United States of America

## Abstract

Evidence suggests the NADPH oxidases contribute to ischaemic stroke injury and Nox2 is the most widely studied subtype in the context of stroke. There is still conjecture however regarding the benefits of inhibiting Nox2 to improve stroke outcome. The current study aimed to examine the temporal effects of genetic Nox2 deletion on neuronal loss after ischaemic stroke using knockout (KO) mice with 6, 24 and 72 hour recovery. Transient cerebral ischaemia was induced via intraluminal filament occlusion and resulted in reduced infarct volumes in Nox2 KO mice at 24 h post-stroke compared to wild-type controls. No protection was evident at either 6 h or 72 h post-stroke, with both genotypes exhibiting similar volumes of damage. Reactive oxygen species were detected using dihydroethidium and were co-localised with neurons and microglia in both genotypes using immunofluorescent double-labelling. The effect of Nox2 deletion on vascular damage and recovery was also examined 24 h and 72 h post-stroke using an antibody against laminin. Blood vessel density was decreased in the ischaemic core of both genotypes 24 h post-stroke and returned to pre-stroke levels only in Nox2 KO mice by 72 h. Overall, these results are the first to show that genetic Nox2 deletion merely delays the progression of neuronal loss after stroke but does not prevent it. Additionally, we show for the first time that Nox2 deletion increases re-vascularisation of the damaged brain by 72 h, which may be important in promoting endogenous brain repair mechanisms that rely on re-vascularisation.

## Introduction

Neuroprotective therapies for the prevention of ischaemic brain injury are an unmet medical need. The relevance of ischaemic stroke is set to escalate with the ageing population, and extensive research efforts have focussed on developing novel therapeutics. Almost without exception, the translation of promising experimental therapeutics into clinically effective treatments has failed. This has highlighted the need for more rigorous pre-clinical testing that includes full investigation of potential therapeutic targets [1], [2].

There are multiple mechanisms responsible for neuronal loss as a result of ischaemic stroke. Oxidative stress occurs due to an imbalance between the generation of reactive oxygen species (ROS), and the antioxidant capacity of the brain. Oxidative stress is thought to be especially relevant during reperfusion after an ischaemic episode: reperfusion provides oxygen as a substrate for ROS generation, which then contributes to lesion progression [3], [4]. The NADPH oxidases are of particular interest in pinpointing the source of oxidative stress, as they are the only enzyme family solely dedicated to the generation of ROS [5]. While numerous antioxidant therapies directed at scavenging reactive species have failed to progress, it is thought that targeting the source of oxidative stress, through NADPH oxidase, could prove a more effective approach to reduce reperfusion injury after stroke.

Several homologues of the NADPH oxidase enzyme have been identified. The first, Nox2 (formerly gp91phox), was originally described in phagocytes [6]. It has now been localised to neurons, blood vessels, microglia, and astrocytes in the brain, in addition to infiltrating inflammatory cells during stroke pathophysiology [7]. The expression of Nox2 and its related regulatory subunits have been shown to increase after stroke at both an mRNA and protein level. ROS generated by increased Nox2 enzyme activity is thought to contribute to vascular inflammation, leukocyte accumulation, oxidative damage, and cell death through multiple pathways, leading to expansion of the ischaemic infarct [7]. Several authors have observed beneficial effects after inhibiting Nox2 in experimental stroke models, reporting decreased infarct volume and improved functional outcome [8]–[13]. However to develop a targeted therapeutic strategy involving Nox2 inhibition, further information about its temporal regulation after stroke, and subsequent contribution to oxidative brain damage is crucial.

In contrast to their widely reported role in infarct evolution in the acute phase of stroke, Nox2-derived ROS are also thought to act as effectors of pro-angiogenic stimuli and may have an important role in brain recovery and regeneration after cerebral ischaemia [14], [15]. It has been shown that Nox2 is beneficial in the peripheral vasculature after hind limb ischaemic injury, contributing to increased capillary density and perfusion, and proliferation of endothelial cells [16], [17]. Additionally, Nox2 was recently co-localised to angiogenic blood vessels in the rat brain up to 7 days post-stroke with reperfusion [18]. Despite these reports, the role of Nox2 in cerebral vascular recovery after stroke in mice is still poorly understood.

We have previously shown a time-dependent increase in Nox2 expression in the rat brain following ischaemia and reperfusion, in association with increased ROS production and progression of injury [19]. In the current study we sought to delineate temporal responses to stroke in mice following genetic deletion of Nox2, including effects on the regenerative capacity of the brain, namely angiogenesis.

## Methods

### Ethics statement

All animal experiments were performed in accordance with the Prevention of Cruelty to Animals Act 1986 under the guidelines of the National Health & Medical Research Council Code for the Care and Use of Animals for Experimental Purposes in Australia 8^th^ edition (2013). The protocol was approved by the St Vincent's Hospital Animal Ethics Committee (AEC 076/08). All surgery was performed under general anaesthesia and included extensive monitoring of each mouse throughout the duration of the study.

### Animals

Knockout mice lacking the Nox2 gene (Nox2 KO) were originally generated in the laboratory of Professor Mary Dinauer [20] and bred at Ozgene (Bentley DC, WA, Australia). Male hemizygous Nox2 KO mice and Nox2 wild-type (Nox2 WT) controls were bred on-site (St Vincent's Hospital EMSU, Fitzroy, VIC, Australia), both maintained on a C57Bl/6 background. A total of 25 Nox2 KO (24–32 g) and 22 Nox2 WT (25–31 g) adult mice (2–3 months) were assigned to one of three groups prior to stroke induction: 6 h, 24 h or 72 h recovery. All mice were housed 4–5 to a cage prior to surgery, on a 12 h day/night cycle at a temperature of 18–22°C with free access to standard chow and water. Post-surgery, mice were housed individually with access to softened food.

### Intraluminal filament middle cerebral artery occlusion

Anaesthesia was induced and maintained through administration of ketamine (100 mg/kg, IP) and xylazine (10 mg/kg, IP), and a heating mat was used to maintain body temperature throughout surgery. As described previously [21], stroke was induced by intraluminal filament occlusion of the right middle cerebral artery (MCA). Briefly, a midline neck incision was made and the right external carotid artery (ECA) was isolated. The superior thyroid artery was cauterised and the ECA was ligated distal to the carotid bifurcation and bisected. The right internal carotid artery (ICA) was isolated and temporarily ligated and the right common carotid artery (CCA) was distally ligated with a microvascular clip. A 6–0 nylon monofilament suture (Doccol Co., Redlands, CA, USA) with a 0.21 mm (for 20–25 g mice) or 0.23 mm (for 25–35 g mice, as per the manufacturer's instructions) silicone tip was introduced into the CCA via an incision in the ligated ECA stump, advanced ∼12 mm distal to the carotid bifurcation and secured in place. Successful occlusion of the MCA (MCAo) was confirmed with laser Doppler flowmetry (Perimed PF 5010; Perimed, Stockholm, Sweden). The ischaemic period was 60 min, during which time the animal remained anaesthetised and body temperature was maintained at 37±0.5°C. At the end of the ischaemic period, the occluding filament was gently withdrawn to allow reperfusion and animals were recovered in a heated cage. A ≥70% decrease in cerebral blood flow (CBF) relative to pre-stroke measurements confirmed effective MCAo. Successful reperfusion was defined as a return to ≥70% of pre-stroke blood flow. Animals that did not meet these criteria were excluded from further analysis. CBF was recorded using PSW 2.5 Light B, 1Ch LDPM software (Perimed).

### Neurological examination

Mice were graded for neurological deficits by a blinded examiner before euthanasia or every 24 h. Mice were scored on two 28 point scales, according to the method of Clark *et al* [22]: the General Score, which examined the general well-being of the animal, including any changes to the hair, ears, eyes, or posture, the level of spontaneous activity, and any epileptic-type behaviour; and the Focal Score, which examined stroke-specific deficits including body symmetry, gait, climbing ability, circling behaviour, front limb symmetry, and whisker response.

### Brain processing

Mice were killed by cervical dislocation and decapitated. The forebrain was quickly removed, frozen over liquid nitrogen and stored at −80°C. Forebrains were later thawed to −18°C and coronal cryostat sections (16 µm) were serially cut at 8 pre-determined planes through the brain (1.98 mm to −3.80 mm relative to bregma), to encompass nuclei from the frontal and parietal cortex and the dorsal and ventral striatum. Sections were then stored at −80°C before further processing.

### Quantification of ischaemic damage

To investigate the effects of Nox2 deletion on stroke damage the infarct was measured in unstained sections using the method described by Callaway *et al* [23]. Using a computerised image analysis system (MCID/M4, Imaging Research Inc., Brock University, St Catharines, ON, Canada), the areas of ischaemic damage were outlined and recorded, while blinded to animal treatment/genotype. Damage to the cortex and subcortex (non-cortical regions) were traced separately. Total infarct volume was calculated by integrating the cross-sectional area of damage at each stereotaxic level with the distances between levels. The influence of oedema on the infarct area was corrected by applying the following formula: (area of normal hemisphere/area of infarcted hemisphere) × area of infarct.

To investigate the effects of Nox2 deletion on neuronal loss a second method of infarct quantification was carried out on sections adjacent to those used for MCID analysis. Images of entire haematoxylin and eosin (H&E) stained sections at each of the 8 levels of interest were acquired using an Aperio ScanScope digital scanner (Aperio Technologies Inc, Vista, CA, USA). Using ImageScope software (Aperio Technologies Inc), infarcted regions of the cortex and subcortex were traced and quantified at 200× magnification through identification of healthy or necrotic neurons, or regions absent of neurons, as per Garcia *et al* [24]. Necrotic/apoptotic neurons were identified as cells exhibiting features such as pyknosis, karyorrhexis, karyolysis, and cytoplasmic eosinophilia or loss of haematoxylin affinity [24]. As per Garcia *et al*, these features can be encompassed under one of two designations: pyknosis/eosinophilia (red neurons) or complete loss of haematoxylinophilia (ghost neurons). Other cellular alterations such as dark, scalloped and swollen neurons were also included in infarct quantification [24]. Total infarct volume was calculated using the same method as employed with MCID-analysed sections, i.e. by integrating the cross-sectional area of damage at each stereotaxic level with the distances between levels.

### Immunofluorescent co-localisation of ROS generation

To identify individual cell types generating ROS, sections were double labelled with dihydroethidium (DHE) and selected antibodies, as described previously [19]. Mouse brain sections were fixed with either cold acetone for neuronal detection, or 4% paraformaldehyde (PFA; Sigma-Aldrich, Castle Hill, NSW, Australia) for inflammatory cell detection. Sections were blocked with serum-free protein block (Dako Australia Pty Ltd, Campbellfield, VIC, Australia) for 30 min. Primary antibody incubations consisted of neuronal mouse monoclonal anti-NeuN (1∶400; Chemicon, Merck Millipore Ltd, Kilsyth, VIC, Australia), macrophage/microglia antibody rabbit anti-Iba1 (1∶1000; Wako Pure Chemical Industries Ltd, Osaka, Japan) or monoclonal rat anti-mouse neutrophil antibody 7/4 (1∶1000; AbD Serotec, Kidlington, UK) in normal donkey serum (NDS; 2%; Sigma-Aldrich), Triton X-100 (0.3%; Sigma-Aldrich) and PBS (0.1 M). Sections were incubated overnight in a humidified chamber at 4°C and then incubated, in the same diluent, with the appropriate secondary antibody: Alexa Fluor 488 donkey anti-mouse IgG (1∶500) for NeuN, Alexa Fluor 488 donkey anti-rabbit IgG (1∶500) for Iba1 or Alexa Fluor 488 donkey anti-rat IgG (1∶500; Invitrogen, Life Technologies Australia Pty Ltd, Mulgrave, VIC, Australia) for neutrophil antibodies. Sections were then double labelled with the ROS indicator, DHE, for co-localisation of ROS and immunofluorescence.

DHE was dissolved in DMSO and further diluted in phosphate buffered saline (PBS, 0.1 M) to a final DMSO concentration of 0.1%, which does not affect ROS generation. Sections were incubated with DHE (2 µM) in a humidified chamber protected from light for 30 min at 37°C. Sections were then rinsed in PBS and cover-slipped with fluorescent mounting medium (Dako Australia Pty Ltd). Analogous negative control experiments were conducted with mouse, rabbit or rat IgG control serum in place of the primary antibody.

Images were taken of regions of interest (core or penumbra), identified using H&E ischaemic quantification images (Zeiss Axioskop2 and Zeiss Axiocam MRc5 with Axiovision software, Carl Zeiss MicroImaging, LLC, Thornwood, NY, USA). The core infarct was defined as damage within the striatum; while the penumbra was defined as the cortex since damage progressed to this region only after 6 hours post-stroke. Core infarct and penumbral tissue were compared to the corresponding contralateral mirror-image region within each animal.

### Laminin immunohistochemistry

An antibody to the basal lamina protein, laminin, was used to visualise blood vessels in sections adjacent to those used for infarct quantification and ROS localisation in 24 h and 72 h recovery groups. Sections were incubated in 3% H_2_O_2_ in PBS to block endogenous peroxidase, fixed with cold acetone and blocked with Dako serum-free protein block. The primary antibody, rabbit polyclonal to laminin (1∶1000; Abcam, Cambridge, UK), was diluted in NDS (2%), Triton X-100 (0.3%) and PBS (0.1 M), and sections were incubated overnight at 4°C. Sections were then incubated with the secondary antibody, Ready to Use Polymer-HRP Conjugate (Invitrogen Australia Pty ltd) for 15 min at room temperature and the antigen was visualised with Liquid DAB Substrate Chromagen System (Dako Australia Pty Ltd).

### Quantification of vessels

Blood vessel density was quantified in laminin-labelled sections by an assessor blinded to recovery group and genotype. For each animal, images of two brain sections at each of the eight levels of interest were acquired with an Aperio ScanScope digital scanner. Using ImageJ, each section was overlaid with a randomly placed grid for point counting, and regions of interest including the infarct core (striatum) and penumbra (cortex) were identified using adjacent H&E images acquired when quantifying infarct volume. For each ipsilateral region of interest and corresponding contralateral mirror-image region, the number of grid points that overlaid laminin-positive blood vessels was counted, and then divided by the total number of grid points within each field. These values were used to assess any difference between genotypes in blood vessel density in the contralateral (control) hemispheres. To assess blood vessel density in the stroke-affected region of interest, ipsilateral values were expressed relative to the corresponding contralateral (mirror-image) value, which is presented as the 100% control.

### Statistical analysis

Neurological deficit scores were analysed using a Kruskal-Wallis non-parametric ANOVA followed by Mann Whitney tests to compare between groups if *P*<0.05. Infarct volume was analysed using paired t-tests and blood flow data and infarct area quantification were analysed using two-way ANOVA. Bonferroni post-hoc tests were used to compare between groups, if *P*<0.05. Infarct volumes are presented as boxplots and include hinges extending from the 25^th^ to 75^th^ percentiles, the median line, and whiskers extending to the minimum and maximum data values. All other data are presented as mean ± standard deviation (SD). A two-sided value of *P*<0.05 was considered statistically significant with power 80%. Differences between ipsilateral vs. contralateral blood vessel density data were analysed using a generalised linear mixed model, and to test whether there was a difference between genotypes, a simple random effects model was fitted. Data were analysed using SigmaPlot 12 (Systat Software Inc., San Jose, CA, USA) or Stata/IC 11.2 for Windows (StataCorp LP, College Station, Texas, USA).

## Results

### Mortality and physiological assessment

A total of 47 mice were used for this study; 13 were excluded from analysis due to premature death outside the desired recovery period, or incomplete reperfusion upon removal of the occluding filament (Table S1). Total mortality was 3/22 for Nox2 WT and 1/25 for Nox2 KO mice; all premature deaths occurred <24 h post-stroke. Only one animal that had adequate reperfusion died; the remainder experienced a ≤70% return to pre-stroke CBF. Some post-stroke weight loss was observed in all stroke groups; however there was no difference between genotypes (data not shown).

### Cerebral blood flow monitoring

Reduction of CBF during MCAo and return of blood flow following reperfusion was confirmed in all animals used for subsequent analyses. There was a significant effect of time on CBF (*P*<0.001, all recovery groups), however no significant effect of genotype was present in 6 h (*P* = 0.576), 24 h (*P* = 0.366) or 72 h (*P* = 0.290) recovery groups (Fig S1), indicating that all animals suffered a similar level of insult.

### Neurobehavioural outcome

No differences existed between genotypes using the general scoring system in the 6 h (*P* = 0.852), 24 h (*P* = 0.172) or 72 h (*P* = 0.238) recovery groups (Fig S2A, C, E). Similarly, no difference existed between genotypes using the focal scoring system in 6 h (*P* = 0.927), 24 h (*P* = 0.399) or 72 h (*P* = 0.160) recovery groups (Fig S2B, D, F). Furthermore, no significant differences were detected between genotypes at any additional time point examined (24 h, 48 h) in the 72 h recovery group (data not shown).

### Quantification of ischaemic damage

**Figure 1:**
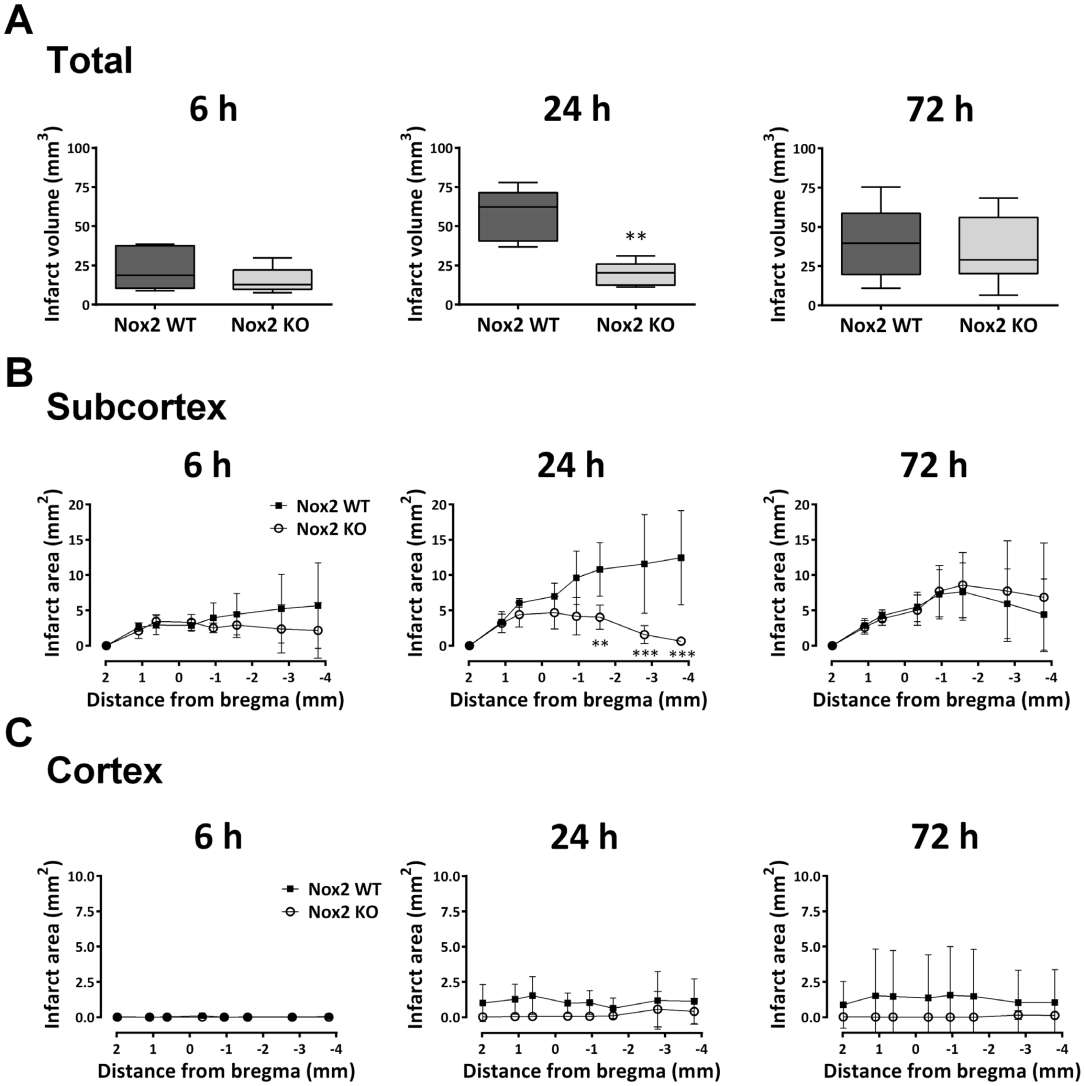
Infarct quantification. Infarct volume was calculated for Nox2 WT and Nox2 KO mice using H&E stained sections after 6 h (n = 5 Nox2 WT, n = 6 Nox2 KO), 24 h (n = 5 per group) or 72 h (n = 5 Nox2 WT, n = 8 Nox2 KO) recovery post-stroke (**A**). Data presented as boxplots; **P<0.01 vs. Nox2 WT, unpaired t-test. The infarct area was calculated in the subcortex (**B**) and cortex (**C**) through the brain at 8 levels relative to bregma in Nox2 WT vs. Nox2 KO mice after 6 h (n = 5 Nox2 WT, n = 6 Nox2 KO), 24 h (n = 5 per group) or 72 h (n = 5 Nox2 WT, n = 8 Nox2 KO) recovery post-stroke. Data presented as mean ±SD. **P<0.01, ***P<0.001 vs. Nox2 WT, Bonferroni tests following two-way ANOVA.

Damage following MCAo was routinely observed in the dorsal and ventral striatum. Other regions affected in some animals included piriform, insular and motor cortex regions as well as subcortical amygdala, thalamus and hippocampus in both genotypes. Data is therefore presented as damage associated with the cortex, and damage associated with non-cortical regions (subcortex). Infarct size was initially quantified in unstained sections using MCID analysis. Total infarct volume was not different between Nox2 WT (12.89±9.65 mm^3^) and Nox2 KO (9.47±5.18 mm^3^) genotypes 6 h post-stroke (*P* = 0.471). At 24 h Nox2 KO mice (15.07±6.98 mm^3^) exhibited significantly less damage than Nox2 WT control mice (35.57±9.53 mm^3^, *P*<0.01), but by 72 h post-stroke, there was again no difference in infarct volumes between genotypes (Nox2 WT 28.77±14.14 mm^3^, Nox2 KO 20.92±9.28 mm^3^, *P* = 0.248). MCID infarct quantification was then validated using H&E stained sections to determine the effects of Nox2 deletion on neuronal loss. Using this method the infarct could be identified at a cellular level, as opposed to the macroscopic changes in tissue appearance used for MCID analysis (*see* Callaway *et al*, 1999). Overall results did not differ between the two methods. Total infarct volume was not different between genotypes at either 6 h (*P* = 0.294) or 72 h (*P* = 0.722) post-stroke, but was reduced in Nox2 KO mice at 24 h post-stroke compared to Nox2 WT controls (*P*<0.01; Fig 1A) using H&E-stained sections. Damage was seen in the subcortex across all recovery times (Fig 1B) while cortical damage was only detected after 24 h or 72 h recovery (Fig 1C). Differences in infarct volume between Nox2 WT mice and Nox2 KO mice at 24 h was due to increased cortical damage anteriorly and subcortical damage posteriorly in Nox2 WT mice (*P*<0.001; Fig 1B, C). Nox2 KO mice were observed to have damage in the posterior subcortex by 72 h (an effect not detected at 24 h; Fig 1B), which resulted in no difference in total infarct volumes between genotypes at this time. A priori power analysis was not carried out for these studies. Given the large difference in means at 24 h, the precision of the estimates was adequate (95% CI 36.70–77.94 Nox2 WT; 9.86–29.00 Nox2 KO) to establish the presence of a genotype effect. However, estimates were less precise at 6 h (95% CI 5.78–40.18 Nox2 WT; 7.10–23.98 Nox2 KO) and 72 h (95% CI 9.93–68.64 Nox2 WT; 17.07–52.33 Nox2 KO). Based on the effects observed in the current experiment, future experiments would need to include n = 38 mice per genotype for 6 h, n = 4 for 24 h and n = 377 for 72 h recovery groups to provide 80% power to detect significant genotype effects at *P* = 0.05.

### Ischaemic damage to regions supplied by the posterior circulation

Given that mice exhibited damage outside the MCA territory, it was important to ensure that one genotype was not unevenly represented for these effects. The number of mice with damage to posterior brain structures including the hippocampus and thalamus was counted. The majority of animals exhibited some posterior cerebral artery (PCA) territory damage within each group (Table S2).

### Immunofluorescent co-localisation of ROS generation

**Figure 2:**
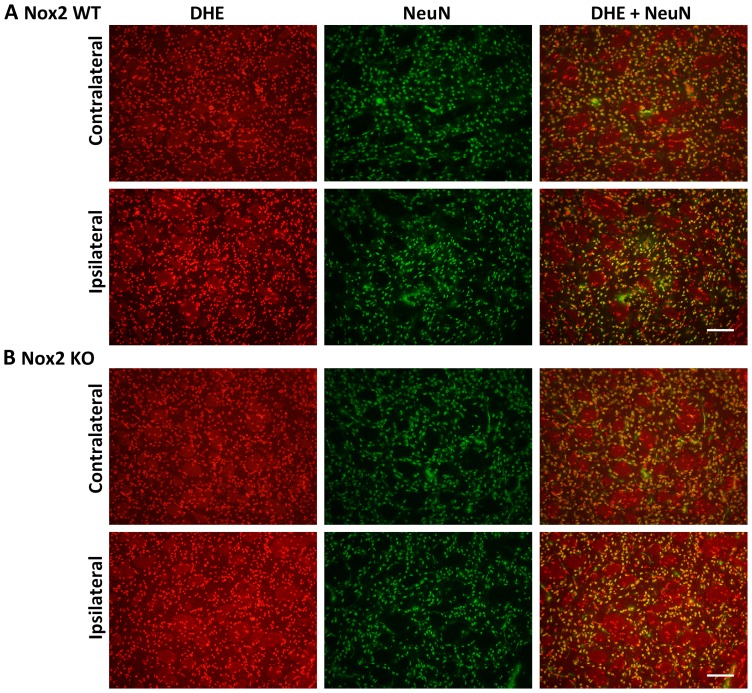
Immunofluorescent co-localisation of ROS generation with NeuN at 6 h. Representative fluorescent micrographs of ROS-sensitive DHE, the neuronal antibody NeuN and merged images from the contralateral and ipsilateral core striatum of Nox2 WT (**A**) and Nox2 KO (**B**) mice at 6 h post-stroke. Scale bars  = 50 µm.

**Figure 3:**
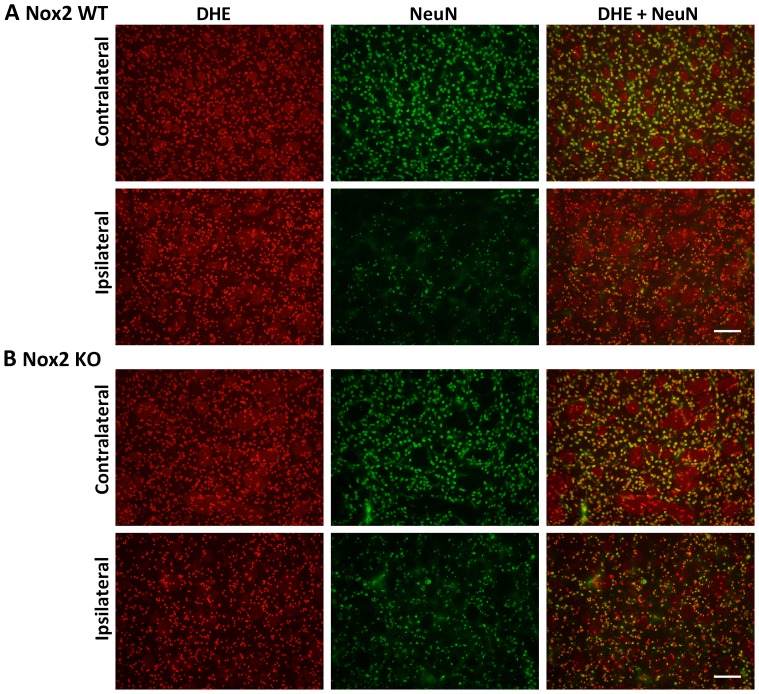
Immunofluorescent co-localisation of ROS generation with NeuN at 24 h. Representative fluorescent micrographs of ROS-sensitive DHE, the neuronal antibody NeuN and merged images from the contralateral and ipsilateral core striatum of Nox2 WT (**A**) and Nox2 KO (**B**) mice at 24 h post-stroke. Scale bars  = 50 µm.

**Figure 4:**
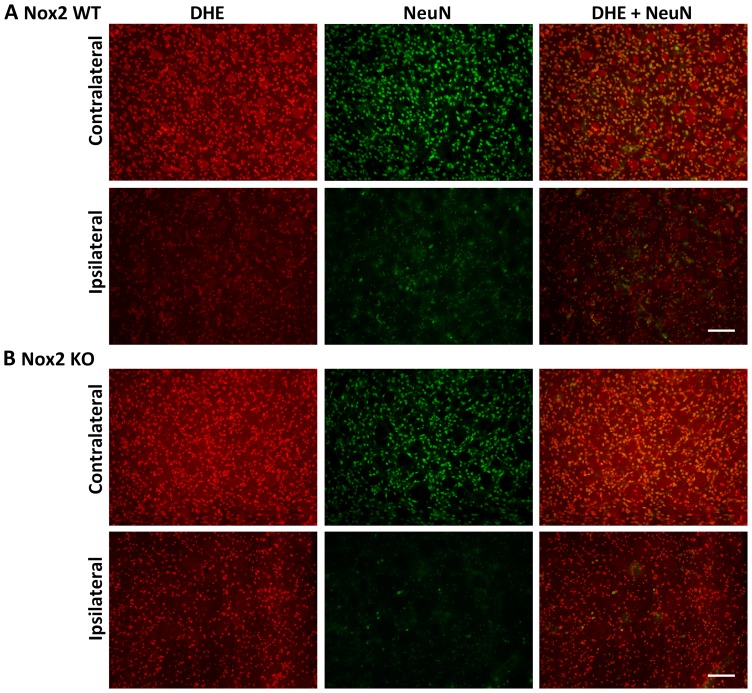
Immunofluorescent co-localisation of ROS generation with NeuN at 72 h. Representative fluorescent micrographs of ROS-sensitive DHE, the neuronal antibody NeuN and merged images from the contralateral and ipsilateral core striatum of Nox2 WT (**A**) and Nox2 KO (**B**) mice at 72 h post-stroke. Scale bars  = 50 µm.

**Figure 5:**
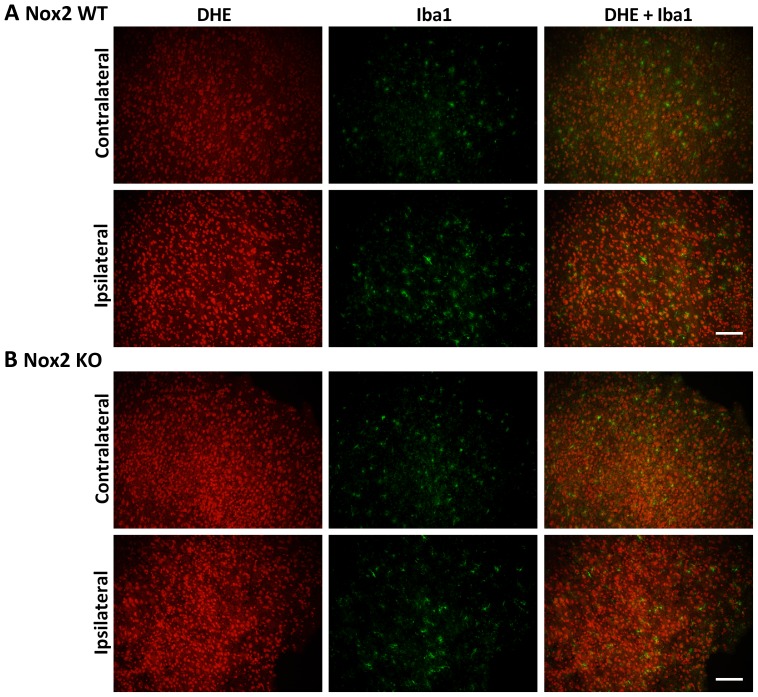
Immunofluorescent co-localisation of ROS generation with Iba1 at 6 h. Representative fluorescent micrographs of ROS-sensitive DHE, the macrophage/activated microglia antibody Iba1 and merged images from the contralateral and ipsilateral penumbral cortex of Nox2 WT (**A**) and Nox2 KO (**B**) mice at 6 h post-stroke. Scale bars  = 50 µm.

**Figure 6:**
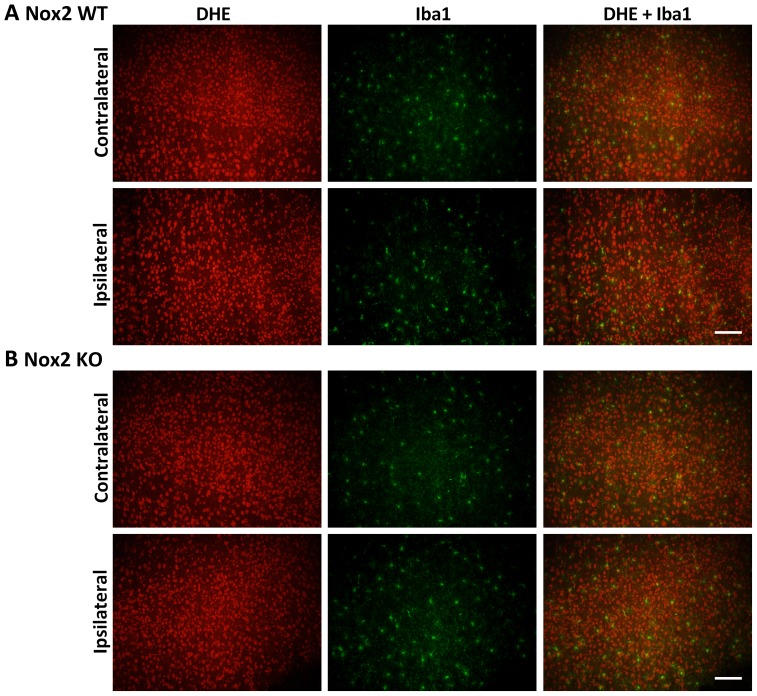
Immunofluorescent co-localisation of ROS generation with Iba1 at 24 h. Representative fluorescent micrographs of ROS-sensitive DHE, the macrophage/activated microglia antibody Iba1 and merged images from the contralateral and ipsilateral penumbral cortex of Nox2 WT (**A**) and Nox2 KO (**B**) mice at 24 h post-stroke. Scale bars  = 50 µm.

**Figure 7:**
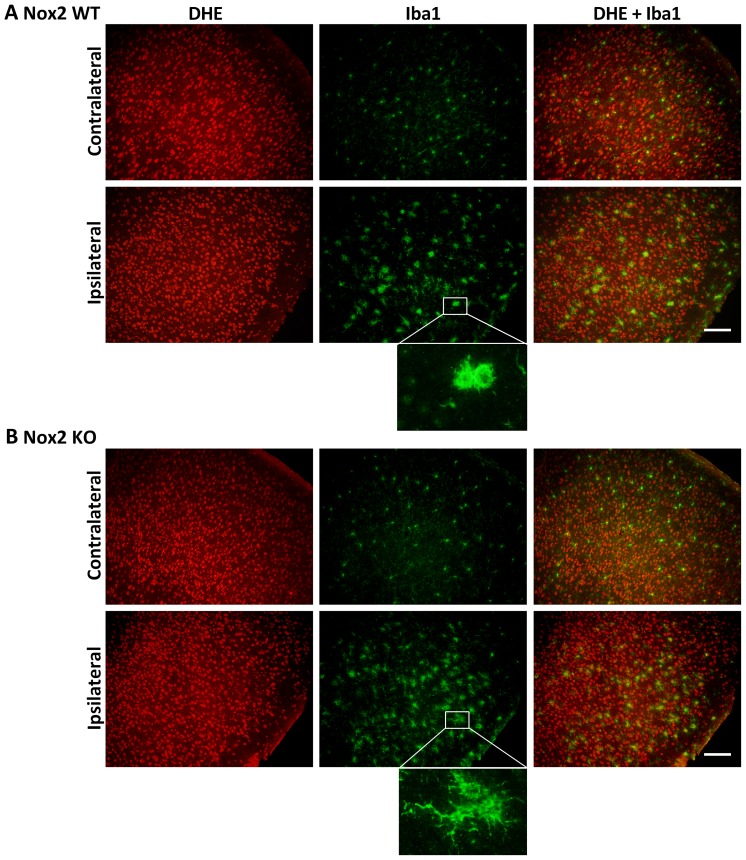
Immunofluorescent co-localisation of ROS generation with Iba1 at 72 h. Representative fluorescent micrographs of ROS-sensitive DHE, the macrophage/activated microglia antibody Iba1 and merged images from the contralateral and ipsilateral penumbral cortex of Nox2 WT (**A**) and Nox2 KO (**B**) mice at 72 h post-stroke. Insets show differing activation states of microglia/macrophages in Nox2 WT vs. Nox2 KO brains. Scale bars  = 50 µm.

Representative images of NeuN and DHE double-labelling are presented for the striatum (core infarct), where the largest change in neuronal DHE fluorescence was observed (Fig 2–4). Images of Iba1 and DHE double-labelling are presented for the cortex (penumbra), where the majority of phagocytic cell immunolabelling occurred (Fig 5–7). Very few neutrophils were detected at any time point examined in either genotype using this model, and their contribution to ROS generation in the current study was not investigated further (images not shown).

Morphological assessment of NeuN-positive cells at 6 h post-stroke revealed cellular changes consistent with necrotic cell death (Fig 2 Ipsilateral), including a shrunken, irregularly shaped nucleus, often triangular in appearance [25]. Most NeuN-positive cells in the striatum at 6 h were also positive for DHE. No prominent changes in morphology were noted in NeuN-positive cells in the cortex (penumbra; images not shown). There were no obvious differences in the number of NeuN and DHE double-labelled cells between genotypes. There was an apparent decrease in the level of DHE-detected ROS in the ipsilateral core over time when compared to the contralateral controls (Fig 2–4). At 24 h and 72 h post-stroke there was a corresponding decrease in the level of NeuN staining, which appeared to be slightly delayed in Nox2 KO mice (Fig 3, 4).

In both genotypes, Iba1 immunoreactivity increased in the ipsilateral penumbra over time. At 6 h post-stroke a low level of activation was observed in the ipsilateral cortex of both Nox2 WT and Nox2 KO mice (Fig 5). These cells were also positive for DHE fluorescence, although Iba1 co-localisation did not appear to account for the majority of DHE signal detected. Iba1-positive labelling in Nox2 WT brain increased in the ipsilateral cortex from 24 h (Fig 6A) to 72 h (Fig 7A) post-stroke, while staining in the contralateral cortex remain unchanged over time. At 72 h post-stroke Iba1-positive cells in the Nox2 KO mice appeared to be less activated (Fig 7B, inset), with cells exhibiting more ramified processes when compared to the amoeboid-like cells observed in Nox2 WT mice (Fig 7A, inset). Iba1-positive cells at all recovery times were co-labelled with DHE in both genotypes. Similar morphological changes in Iba1-positive cells were observed in the striatum at all recovery times but the degree of activation appeared to be less than that seen in the cortex (images not shown).

### Quantification of blood vessel density

**Figure 8:**
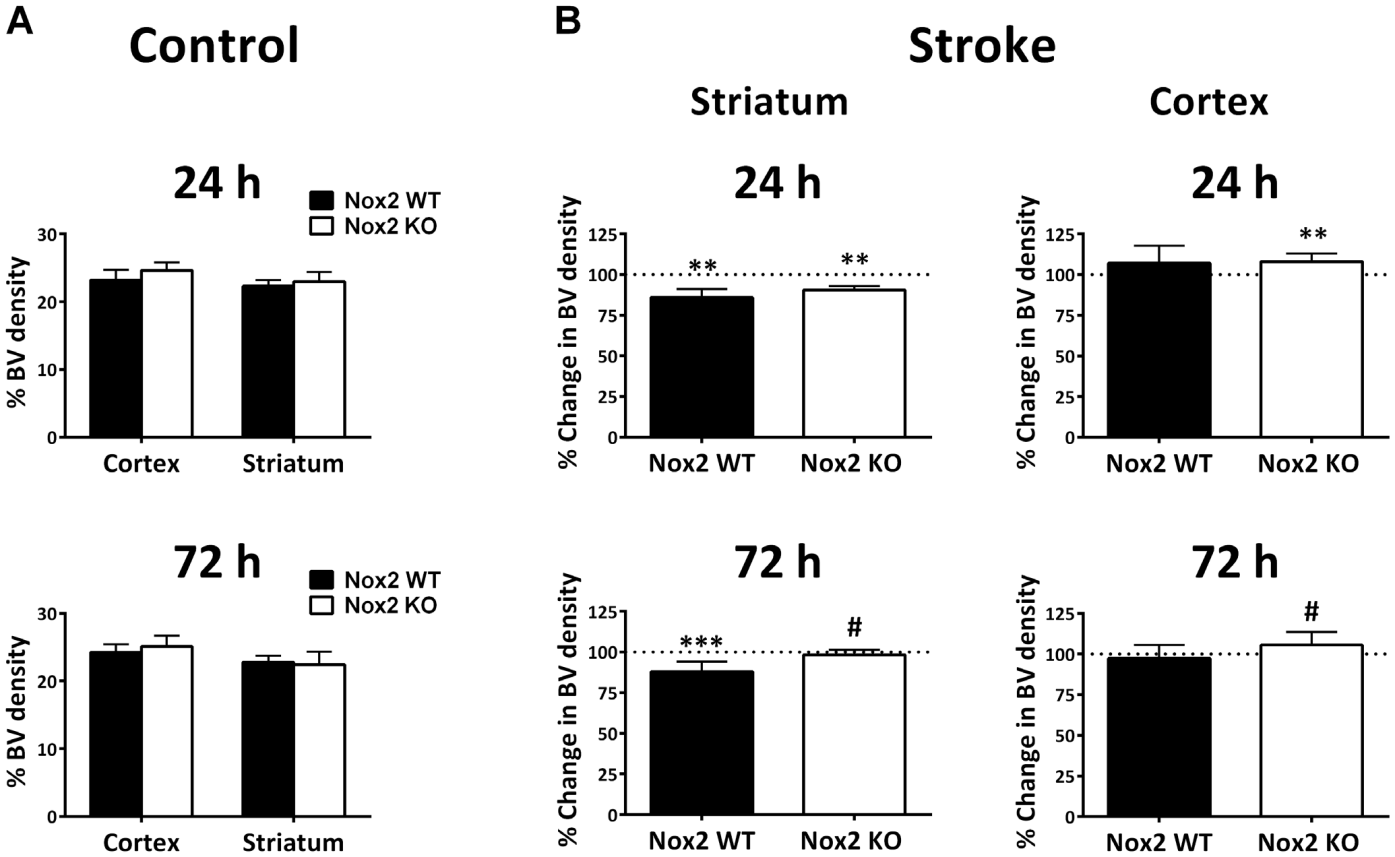
Quantification of blood vessel density. Blood vessel (BV) density was first quantified in the contralateral (control) hemisphere in the cortex and striatum of Nox2 WT and Nox2 KO mice at 24 h and 72 h post-stroke (**A**). Blood vessel density was then quantified in the stroke-affected striatum (core infarct) and cortex (penumbral region) at 24 h (n = 5 per group) and 72 h (n = 5 Nox2 WT, n = 8 Nox2 KO) recovery (**B**). Blood vessel density in the ipsilateral (stroke-affected) hemisphere is expressed as a percentage of that in the corresponding contralateral (non-affected; 100% control, dotted line) hemisphere. Data presented as mean +SD. **P<0.01, ***P<0.001 vs. contralateral control (100%), generalised linear mixed model; # *P*<0.05 vs. Nox2 WT, simple random effects model.

Blood vessel density in the contralateral (control) cortex and striatum at 24 h and 72 h post-stroke was quantified as a measure of the normal blood vessel density in both Nox2 WT and Nox2 KO mouse brains. There was no effect of genotype on blood vessel density at 24 h (*P* = 0.080) or 72 h (*P* = 0.655) post-stroke (Fig 8A).

The density of blood vessels in the stroke-affected region was then expressed as a percentage of that in the corresponding contralateral mirror-image region to determine the effects of stroke damage on blood vessel density. In the ipsilateral striatum, blood vessel density decreased in both Nox2 WT (86.0±10.3%, *P*<0.01) and Nox2 KO (90.4±5.8, *P*<0.01) mice at 24 h when compared to the contralateral control region (100% control; Fig 8B). At 72 h post-stroke, blood vessel density remained decreased in the ipsilateral striatum in Nox2 WT mice (88.0±7.7%, *P*<0.001), but returned to control levels in Nox2 KO mice (98.3±7.0%, *P* = 0.256; Fig 8B). This resulted in a significant difference between genotypes in the change in core blood vessel density compared to the corresponding contralateral control values at 72 h (*P*<0.05). Blood vessel density in the ipsilateral cortex (penumbra) of Nox2 KO animals was significantly increased 24 h after stroke when compared to the contralateral control (108.2±4.3%, *P*<0.01; Fig 8B). While there was a trend towards increased ipsilateral blood vessel density in Nox2 WT animals at 24 h, no significant difference existed compared to control (106.4±6.5%, *P* = 0.111) and there was no difference between genotypes (*P* = 0.725; Fig 8B). By 72 h post-stroke, no difference in blood vessel density in the ipsilateral compared to the contralateral cortex was observed in either Nox2 WT (97.4±6.5%, *P* = 0.251) or Nox2 KO (105.6±6.7%, *P* = 0.363) mice, however there was a difference between genotypes (*P*<0.05; Fig 8B).

## Discussion

There are conflicting reports regarding the influence of Nox2 on brain injury after stroke. Here we have included within the same study design multiple recovery times to investigate the effects of Nox2 deletion on infarct progression. Furthermore, we have extended these studies to investigate the effect of Nox2 deletion on early vascular loss and subsequent recovery after stroke. This is the first study where multiple recovery times are used to report an initial protective effect of Nox2 deletion at 24 hours that is not evident at 72 hours. These results suggest that brain injury is merely delayed in mice lacking a functional Nox2. We also report for the first time that Nox2 deletion does not affect cerebral vascular loss after stroke but it does improve vascular recovery by 3 days. These results collectively suggest that although not effective in preventing long-term neuronal injury, Nox2 may still be an important target for promoting early re-vascularisation to support brain repair.

### Infarct progression and functional outcome

Previous studies have detailed neuroprotection by genetic deletion of Nox2 at both 24 h [10], [26]–[28] and 72 h [26], [27], [29] post-stroke. However, others have also reported a lack of neuroprotection with Nox2 deletion at 24 h [30]. Additionally, pharmacological inhibition of NADPH oxidase does not reduce the extent of brain injury caused by hypoxia-ischaemia in perinatal mice [31] and Nox2 KO mice show no reduction in injury or mortality 24 h after haemorrhagic stroke [32]. To our knowledge, no published data exist detailing recovery beyond 72 h post-stroke in a Nox2 KO strain; the difficulty recovering mice for extended periods has presumably prohibited such studies [33]. Similarly, the long term efficacy of pharmacological agents used to inhibit Nox2 has not been investigated to date. One of the most widely studied Nox2 inhibitors, apocynin, has no effect on stroke size if administered post-stroke [9], [30] and is ineffective in aged rats [34]. Many published studies only assess the effects of apocynin pre-treatment at 24 h after stroke, in both rat and mouse focal ischaemia models [9], [11], [12], [35], [36]. Studies in our laboratory recently extended this treatment period and showed that apocynin had no effect on oxidative stress at 3 days post-stroke, indicating that it is unlikely that Nox2-derived ROS are involved in lesion progression at this time [13]. Other pharmacological agents that inhibit the Nox2 oxidase, including atorvastatin and betulinic acid, also require pre-treatment to be effective, and again, outcomes are reported only at 24 h post-stroke [8], [37]. Thus, the efficacy of pharmacological agents that target Nox2, when administered within a timeframe likely to be clinically relevant, is also yet to be proven. In the present study we confirm the increase in neuronal loss between 24 h and 72 h after stroke in Nox2 KO mice using H&E staining. The low precision in 6 h and 72 h group infarct volume data suggest that these results be interpreted with some caution due to an increased potential for Type II error. We calculated that 377 mice per group would be needed in future experiments to detect significance in the genotype effect observed at 72 h post-stroke. However the difference in means between genotypes was <12% (based on the more detailed H&E infarct quantification), a change smaller than that usually sought in preclinical stroke studies. Together with the above reports, the current results suggest that targeting Nox2 for long-term neuroprotection is unlikely to translate to clinical success.

It is interesting to note that Nox2 is abundantly expressed on the cell membrane of inflammatory cells, which are thought to play an important role in the pathophysiology of stroke beyond the acute phase. However, studies investigating long-term stroke outcomes have found that although compounds targeting inflammation can be effective with a short survival period, these initial benefits may be lost over time [38], [39]. This phenomenon is not restricted to anti-inflammatory drugs: the NMDA receptor antagonist, MK-801, was found to be neuroprotective at 3 days, but no significant difference in infarct size between treated and placebo rats was evident 28 days after MCAo [40]. Importantly, the level of neuroprotection observed in Nox2-deficient mice reported by Chen *et al* [26] decreased by 72 h when compared to the 24 h recovery group. This furthers speculation that Nox2 inhibition may not provide lasting neuroprotection and indicates that studies need to be repeated across multiple groups with longer recovery periods post-stroke.

### Regional specific damage

The use of the thread occlusion model to induce MCAo is known to often generate damage outside of the MCA territory and may account for the discrepancies in outcomes previously reported in Nox2 KO studies. In the present study the lesion encompassed brain regions supplied not only by the MCA, but also regions supplied primarily by the PCA, including the hippocampus and thalamus [41]. Damage to the PCA territory is thought to be linked to vascular abnormalities including the absence of one or both posterior communicating arteries (PcomA) [41], [42]. The C57Bl/6 mouse strain, used as the background strain in the current study, is widely used in the generation of genetically modified mice and is reported to often exhibit these abnormalities [41]–[43]. To our knowledge, we are the first to fully describe all brain regions affected when using the filament occlusion model in Nox2 KO mice and as such have made appropriate comparisons across groups. The difference in infarct volume between the genotypes in the current study at 24 h appeared to be predominantly due to more damage in the posterior areas of Nox2 WT mouse brains, encompassing both MCA and PCA territories. Hence we investigated whether there was a difference in the proportion of animals within each genotype that had damage to the PCA territory, and were therefore likely to be lacking perfusion through the PcomA. While the extent of damage to the PCA territory was found to differ between the genotypes, an equal proportion of animals from each genotype exhibited some degree of PCA territory damage within each recovery group, and damage in the PCA territory at 72 h was not significantly different between groups. This indicates that abnormalities in mouse vasculature most likely did not contribute to the observed differences between genotypes. However, analysis of the Circle of Willis anatomy in both genotypes would be required to confirm this finding.

### Contribution of Nox2 to ROS production post-stroke

The cell type that contributes most significantly to injury-induced ROS production in the brain is still unclear. We have previously reported an increase in neuronal ROS at 6 h post-stroke following ischaemia and reperfusion in the rat brain [19]. In the current study, ROS generation was again co-localised with neurons at 6 h post-stroke in both genotypes, indicating that Nox2 is not the sole contributor to oxidative stress after stroke in this model; other sources of ROS are also active and may contribute to lesion development. Additional sources of ROS at 6 h may include cyclooxygenases, lipoxygenases, xanthine oxidase, or other NADPH oxidase subtypes. Indeed Nox4 is reported to be increased in neurons following both experimental and clinical stroke and to be an important regulator of focal ischaemic stroke in the mouse [30]; it may contribute to ROS generation and lesion progression in the current study.

While the majority of cells positive for DHE appeared to be neurons in both genotypes at 6 h, DHE was also detected in activated microglia. In agreement with Chen *et al* [26], microglia from Nox2 KO mice appeared to be less activated than those from Nox2 WT mice. This was evidenced by a more ramified appearance with longer processes in Nox2 KO microglia, compared with the amoeboid appearance of Nox2 WT microglia. It was interesting to note that despite lacking Nox2, believed to be the predominant source of superoxide in microglia and macrophages, Iba1-positive cells from Nox2 KO mouse brains show extensive double-labelling with DHE. In addition to Nox2, microglia have also been reported to express both Nox1 and Nox4 [44]–[47]. Employing a mouse model of myocardial infarction, Zhao *et al* [48] reported that during NADPH oxidase deficiency, compensatory superoxide production from alternative sources occurred. This led to cardiac oxidative stress and no difference in myocardial infarct size between Nox2 KO mice and wild-type littermates. Additionally, up-regulation of alternate Nox subtypes in genetically modified strains has been reported [49], [50]. Indeed this is a concern with most genetically modified models: unexpected phenotypes can occur due to the absence of a gene during the entire lifespan of an animal [51].

### Effect of Nox2 inhibition on cerebral blood vessel density

Nox2 is highly expressed in cerebral arteries and plays an important role in cerebrovascular superoxide generation [52]. We have recently shown that changes in vascular density after stroke in rats correlates with increased expression of Nox2 in angiogenic vessels [13]. To our knowledge, changes in cerebral blood vessels early after stroke in Nox2 KO mice have not previously been reported, nor has the role of Nox2 in relation to vascular recovery after stroke in mice been investigated. We found no differences between genotypes in the detection of blood vessel density in the non-damaged hemisphere, suggesting that Nox2 deletion in this model does not affect normal brain vascular density during development. The decrease in core blood vessel density detected in both genotypes 24 h after stroke is consistent with vascular loss in response to stroke as reported by others [53]. However by 72 h post-stroke, vascular density appeared to have recovered in Nox2 KO mice, indicating that while Nox2 deletion does not confer long term protection on neurons in the core, it may be beneficial in terms of restoring the cerebral vasculature to pre-stroke levels. It remains unclear whether the absence of Nox2 activity within the vessels themselves is beneficial, or if the absence of Nox2 from surrounding cells creates an environment more conducive to new vessel growth. Compensatory changes in other NADPH oxidases may also be involved in the observed effects, with Nox4 reported to play a significant role in angiogenesis [54].

Angiogenesis is thought to be essential for ischaemic brain repair as it stimulates blood flow and metabolism in the ischaemic boundary, and may provide critical neurovascular substrates for neuronal remodelling [55]. Proliferating endothelial cells, indicative of angiogenesis, have been shown to increase as early as 24 h after stroke in a mouse MCAo model [56]. We have previously shown the importance of Nox2 in promoting cell survival during rat endothelial cell proliferation *in vitro* and that inhibition of Nox2 significantly suppresses vessel growth [56]. Others have reported that perfusion recovery and an increase in capillary density are significantly inhibited in Nox2 KO mice at 7–14 days after ischaemic hind limb injury. The current study is the first to show that Nox2 deletion does not affect vascular loss after stroke but does result in increased vascular staining in the damaged brain by 3 days. This apparent beneficial effect of Nox2 deletion on vascular recovery may reflect the early time point examined. Alternatively this may be a lasting effect, reflecting the differential expression and roles of the NADPH oxidases in the cerebral vs. systemic vasculature.

## Conclusions

There is a substantial body of research which suggests that blocking the production of ROS by targeting a responsible enzyme may deliver a more favourable therapeutic outcome after ischaemic stroke with reperfusion. The NADPH oxidases present such a target, however the transient nature of the protection afforded by Nox2 deletion in the current study suggests that other factors may be of greater significance in the search for acute therapies. While there is evidence to show that Nox2 is harmful in the acute phase of stroke, the results of the present and previous studies suggest that it may merely delay infarct progression, and not prevent it. Of particular interest in the current study is the potential to target Nox2 for promoting early re-vascularisation after stroke. Therapeutics aimed at manipulating Nox2 may improve brain repair and subsequent long term functional recovery, the ultimate goal of any stroke therapy.

## Supporting Information

Figure S1 **Change in cerebral blood flow (CBF) during stroke induction.** Change in CBF for Nox2 WT and Nox2 KO mice was recorded using laser Doppler flowmetry for 6 h (**A**; n = 5 Nox2 WT, n = 6 Nox2 KO), 24 h (**B**; n = 5 per group) and 72 h (**C**; n = 5 Nox2 WT, n = 8 Nox2 KO) recovery groups. Data are presented for the ischaemic period (0–60 min) and subsequent reperfusion (>60 min) as change in CBF from pre-stroke values (100%). Data presented as mean ±SD. There was no effect of genotype on CBF in any recovery group, RM two-way ANOVA.
(TIF)

Figure S2 **Neurological deficit scores.** General (**A**, **C**, **E**) and focal (**B**, **D**, **F**) neurological deficit scores for 6 h (**A**, **B**), 24 h (**C**, **D**) and 72 h (**E**, **F**) post-stroke recovery groups. Only the final neurological deficit scores taken for each group are presented. Data presented as scatterplots overlaid with the median and IQR; no significant differences between groups were detected at any time point, Mann Whitney tests.
(TIF)

Table S1 **Mice excluded from analysis.**
(TIF)

Table S2 **Proportion of mice with damage outside the MCA territory.**
(TIF)
