# Supplementary figures and images for: Nox2 Knockout Delays Infarct Progression and Increases Vascular Recovery through Angiogenesis in Mice following Ischaemic Stroke with Reperfusion
Source: PLoS One. 2014 Nov 6;9(11):e110602. doi: 10.1371/journal.pone.0110602 (PMC4222846; doi:10.1371/journal.pone.0110602)

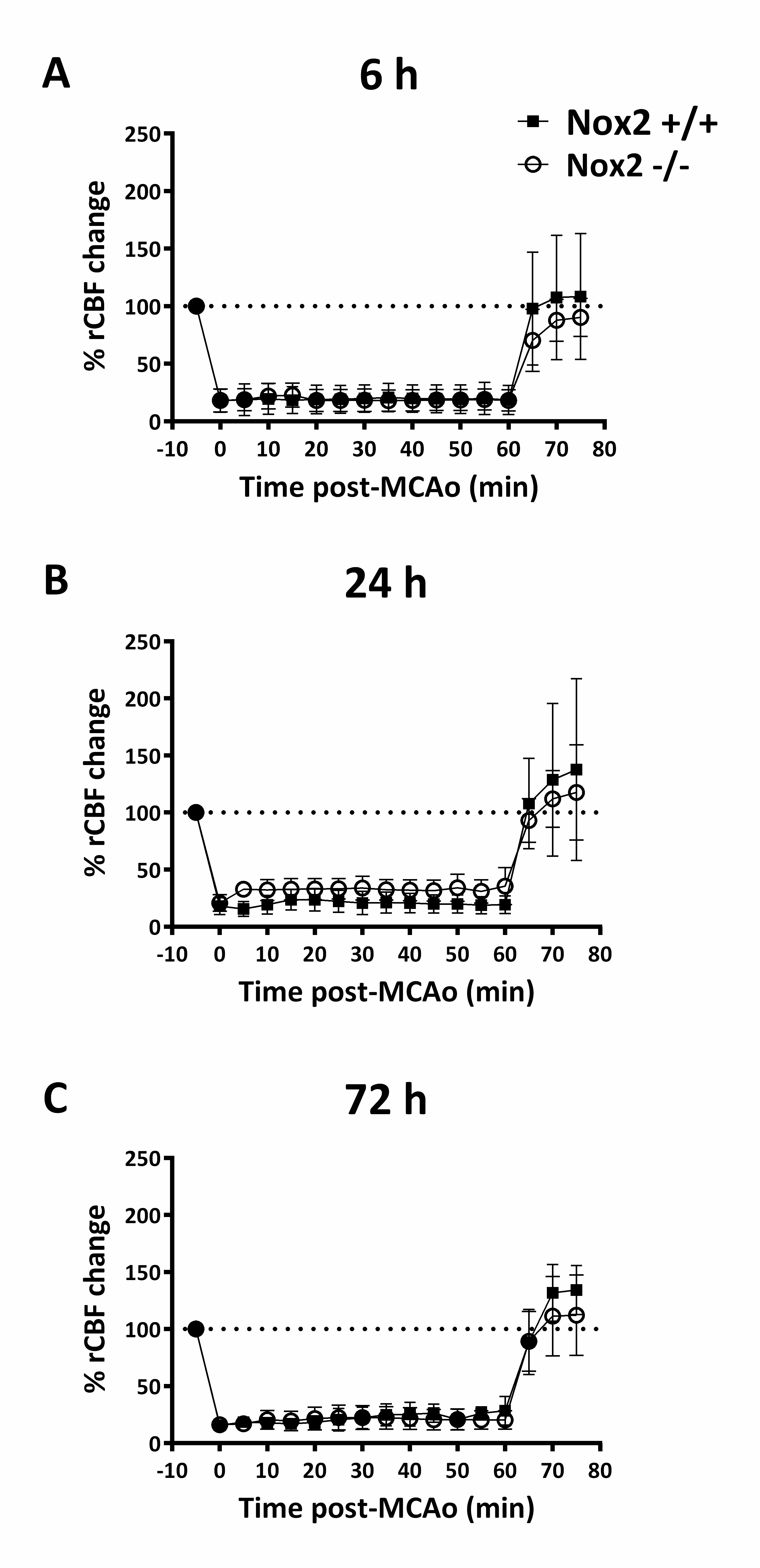

Supplement: Figure S1 — Change in cerebral blood flow (CBF) during stroke induction. Change in CBF for Nox2 WT and Nox2 KO mice was recorded using laser Doppler flowmetry for 6 h (A; n = 5 Nox2 WT, n = 6 Nox2 KO), 24 h (B; n = 5 per group) and 72 h (C; n = 5 Nox2 WT, n = 8 Nox2 KO) recovery groups. Data are presented for the ischaemic period (0–60 min) and subsequent reperfusion (>60 min) as change in CBF from pre-stroke values (100%). Data presented as mean ±SD. There was no effect of genotype on CBF in any recovery group, RM two-way ANOVA. (TIF) [file pone.0110602.s001.tif]

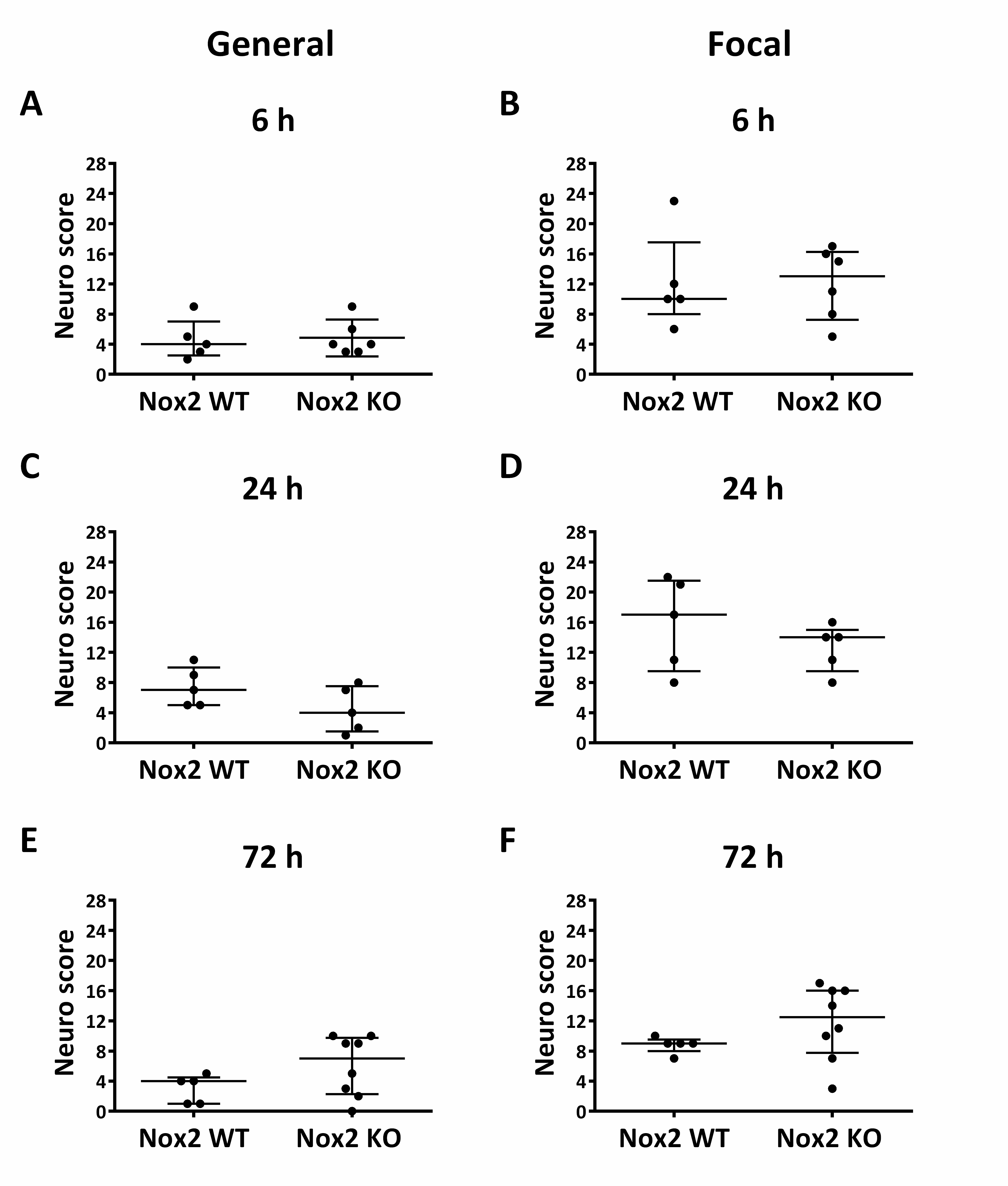

Supplement: Figure S2 — Neurological deficit scores. General (A, C, E) and focal (B, D, F) neurological deficit scores for 6 h (A, B), 24 h (C, D) and 72 h (E, F) post-stroke recovery groups. Only the final neurological deficit scores taken for each group are presented. Data presented as scatterplots overlaid with the median and IQR; no significant differences between groups were detected at any time point, Mann Whitney tests. (TIF) [file pone.0110602.s002.tif]

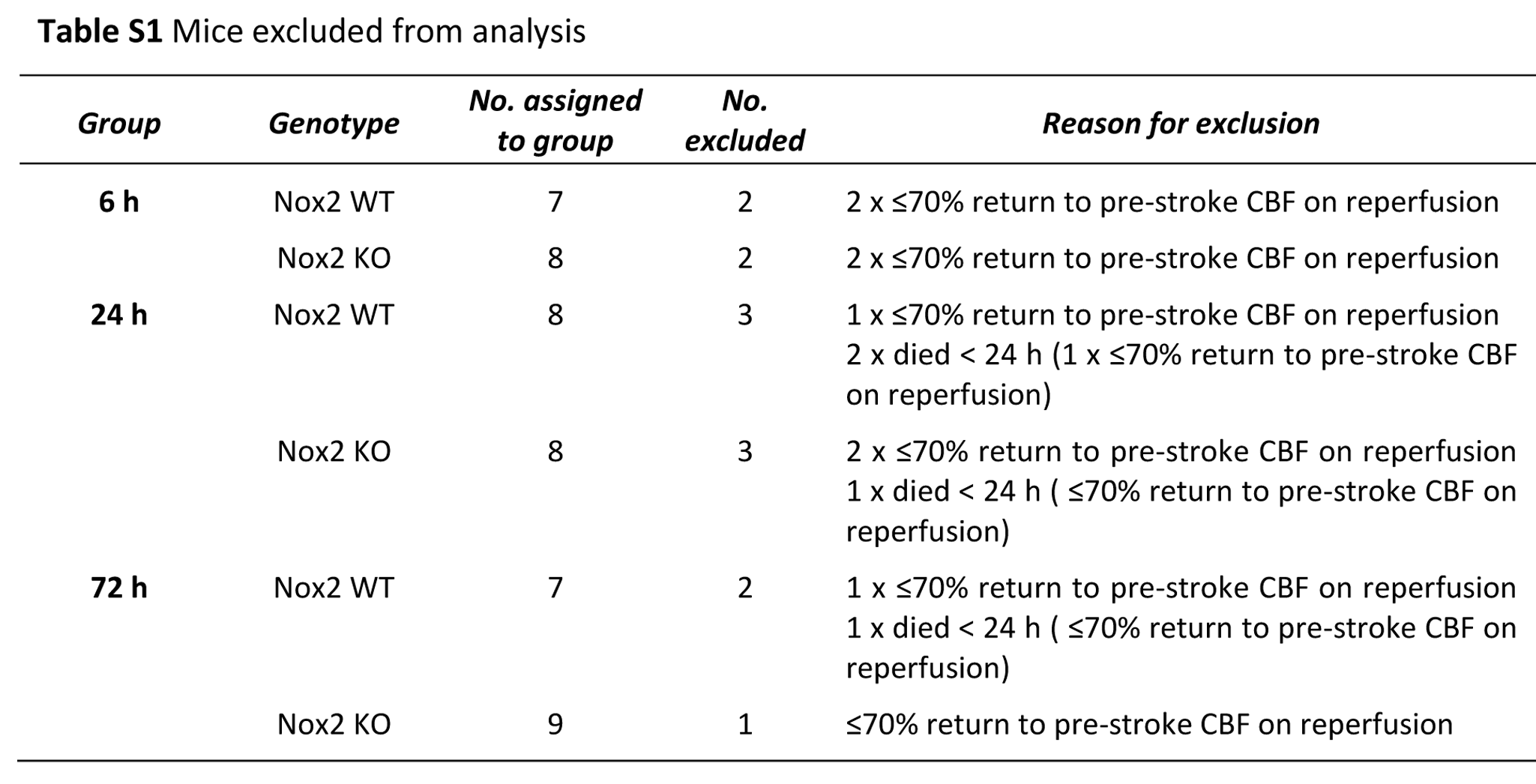

Supplement: Table S1 — Mice excluded from analysis. (TIF) [file pone.0110602.s003.tif]

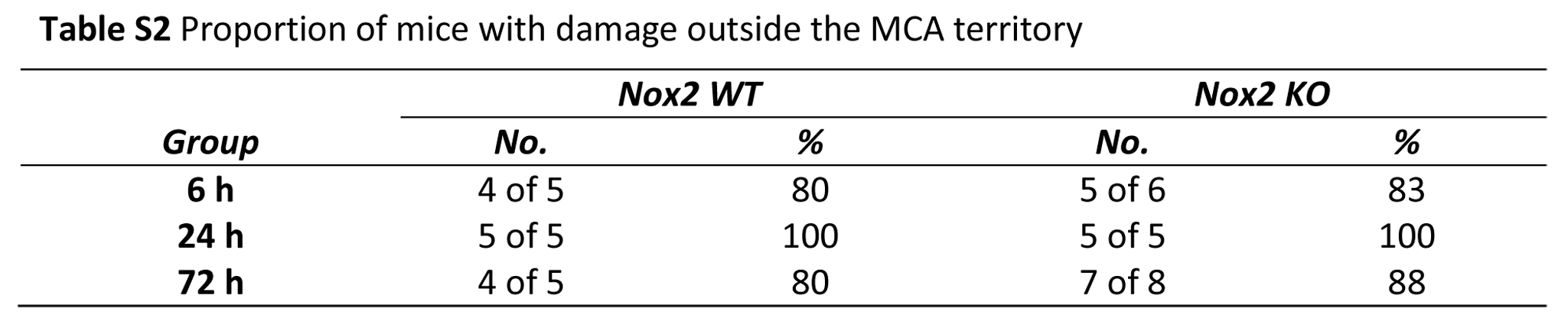

Supplement: Table S2 — Proportion of mice with damage outside the MCA territory. (TIF) [file pone.0110602.s004.tif]
